# DFP: a Bioconductor package for fuzzy profile identification and gene reduction of microarray data

**DOI:** 10.1186/1471-2105-10-37

**Published:** 2009-01-29

**Authors:** Daniel Glez-Peña, Rodrigo Álvarez, Fernando Díaz, Florentino Fdez-Riverola

**Affiliations:** 1 Escuela Superior de Ingeniería Informática, University of Vigo, Edificio Politécnico, Campus Universitario As Lagoas s/n, 32004 Ourense, Spain; 2 Departamento de Informática, University of Vigo, Edificio Fundición, Campus As Lagoas-Marcosende, 36310 Vigo, Pontevedra, Spain; 3 Escuela Universitaria de Informática, University of Valladolid, Plaza Santa Eulalia, 9-11, 40005 Segovia, Spain

## Abstract

**Background:**

Expression profiling assays done by using DNA microarray technology generate enormous data sets that are not amenable to simple analysis. The greatest challenge in maximizing the use of this huge amount of data is to develop algorithms to interpret and interconnect results from different genes under different conditions. In this context, fuzzy logic can provide a systematic and unbiased way to both (*i*) find biologically significant insights relating to meaningful genes, thereby removing the need for expert knowledge in preliminary steps of microarray data analyses and (*ii*) reduce the cost and complexity of later applied machine learning techniques being able to achieve interpretable models.

**Results:**

DFP is a new Bioconductor R package that implements a method for discretizing and selecting differentially expressed genes based on the application of fuzzy logic. DFP takes advantage of fuzzy membership functions to assign linguistic labels to gene expression levels. The technique builds a reduced set of relevant genes (FP, *Fuzzy Pattern*) able to summarize and represent each underlying class (pathology). A last step constructs a biased set of genes (DFP, *Discriminant Fuzzy Pattern*) by intersecting existing fuzzy patterns in order to detect discriminative elements. In addition, the software provides new functions and visualisation tools that summarize achieved results and aid in the interpretation of differentially expressed genes from multiple microarray experiments.

**Conclusion:**

DFP integrates with other packages of the Bioconductor project, uses common data structures and is accompanied by ample documentation. It has the advantage that its parameters are highly configurable, facilitating the discovery of biologically relevant connections between sets of genes belonging to different pathologies. This information makes it possible to automatically filter irrelevant genes thereby reducing the large volume of data supplied by microarray experiments. Based on these contributions GENECBR, a successful tool for cancer diagnosis using microarray datasets, has recently been released.

## Background

Microarray techniques have revolutionized genomic research by making it possible to monitor the expression of thousands of genes in parallel. Due to the amount of data being produced by this technology, gene reduction is extremely important because: (*i*) it generally reduces the computational cost of machine learning techniques, (*ii*) it usually increases the accuracy of classification algorithms and (*iii*) it provides clues to researches about genes that are important in a given context (i.e. biomarkers for certain diseases, etc.) [1].

Related with this domain, the area of gene identification has been previously addressed by Furman *et al*. through the utilization of information theory [2]. Several methods have been proposed to reduce dimensions in the microarray data domain. These works include the application of genetic algorithms [3], wrapper approaches [4], support vector machines [5,6], spectral biclustering [7], etc. Other approaches focus their attention on redundancy reduction and feature extraction [8,9], as well as the identification of similar gene classes making prototypes-genes [10].

In addition, there are also several packages implemented in R for feature selection as iterativeBMA [11], varSelRF [12,13] or R-SVM [14]. iterativeBMA is a Bioconductor R package which performs multivariate feature selection for multiclass microarray data and it is based on the bayesian model averaging (BMA) approach. The varSelRF package implements a method for gene selection based on the measures of variable importance which return the random forest algorithm and it is also suitable for multivariate and multiclass datasets. The R-SVM method is similar to the varSelRF in the sense that it uses the relative importance of features in SVM classifiers to select relevant genes but it is only applicable to binary classifications. Finally, it is also considered the ttest function of the genefilter package (available from Bioconductor) which implements the conventional t-test method for feature selection. Table 1 shows a comparative analysis of these R-based methods and the proposed DFP algorithm.

**Table 1:** Comparative analysis of R-based methods for gene selection

	**iterativeBMA **[11]	**varSelRF **[12,13]	**R-SVM **[14]	**ttest **[genefilter]	**DFP**
Method	Bayesian model averaging (BMA) approach over the underlying classification model (logistic regression)	varSelRF uses the measures of variable importance (related to the classification) provided directly by the Random Forest algorithm	R-SVM uses a contribution factor of each feature (computed from the weights of the SVM classifier)	t-test	The selected genes are based on the induced fuzzy pattern for each class
Type of classification	Multiclass	Multiclass	Binary classifications	Binary classifications	Multiclass
Dependence among features	Multivariate	Multivariate	Multivariate	Univariate	Univariate
Remarks	The method facilitates biological interpretation by producing posterior probabilities of selected genes and models. BMA accounts for the uncertainty about the best set to choose by averaging over multiple models The R package is available from Bioconductor The method requires a limit in the maximum number of relevant genes to be selected and the final results are conditioned by an initial selection based on a univariate gene selection method	The method does not require pre-specify the number of genes to be selected, but rather adaptively chooses the number of genes The R package is available from CRAN and its implementation takes advantage of computing clusters and multicore processors The varSelRF is biased to identify small sets of genes that can still achieve good predictive performance (thus, highly correlated genes will not be selected since they are considered as redundant genes)	The algorithm is based on the repeated application of the SVM classifier over progressively smaller sets of genes (where genes are excluded according to the defined contribution factor) until a satisfactory solution is achieved. The number of iterations and the number of features to be selected in each iteration are very *ad hoc* The R-SVM method is only suitable for binary classifications	The computational effort is smaller than multivariate methods The genefilter package is available from Bioconductor It is sensitive against outliers which are frequent in microarray data It requires normal distribution of the expressions levels within both classes	It does not require any assumption about the distribution of the expression levels and It accounts for the noise in the data because, as a fuzzy-based method, it deals with linguistic categories instead of raw data The implementation is computationally efficient and available from Bioconductor The DFP method does not take into consideration that features are influencing a biological outcome in the context of networks of interacting genes

In this context, there are many advantages of applying fuzzy logic to the analysis of gene expression data: (*i*) fuzzy logic inherently accounts for noise in the data because it extracts trends, not crisp values; (*ii*) in contrast to other automated decision making techniques, algorithms in fuzzy logic are cast in the same language used in day-to-day conversation, so conclusions are easily interpretable and can be extrapolated; (*iii*) fuzzy logic techniques are computationally efficient and can be scaled to include a high number of components [15].

Based on these assumptions, the aim in writing DFP was to provide a simple-to-use library to perform gene selection and data reduction by the application of a supervised fuzzy pattern algorithm able to discretize and filter existing gene expression profiles.

## Implementation

DFP is an extension package for the programming language and statistical environment R [16]. The software has been developed to perform fuzzy analysis and gene reduction using microarray data. It employs object classes and functions that are also standard in other packages of the Bioconductor project [17]. The whole algorithm comprises of three main steps. First, it represents each gene value in terms of one from the following linguistic labels: Low, Medium, High and their intersections LowMedium and MediumHigh. The output is a *fuzzy microarray descriptor *(FMD) for each existing sample (microarray) containing the discretized gene expression values. The second phase aims to find all genes that best explain each class, constructing a supervised *fuzzy pattern *(FP) for each class (pathology). Starting from the previous generated fuzzy patterns, the package is able to discriminate those genes that can provide a substantial discernibility between existing classes, generating an unique *discriminant fuzzy pattern *(DFP).

### Discretizing microarray data using fuzzy labels

**Figure 1:**
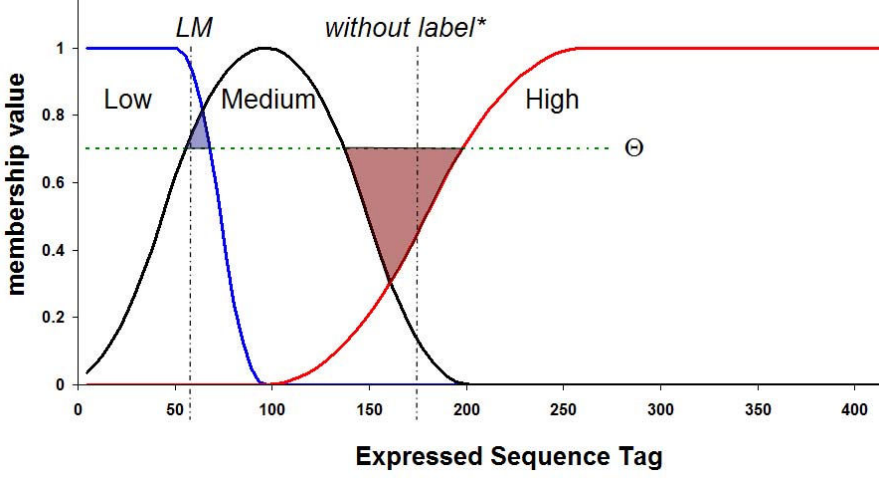
**Shape of membership function for a specific gene and possible assigned labels given a threshold θ = 0.7**. The centre and amplitude of each membership function depend on the mean and on the variability of the available data respectively. The Medium membership function is considered symmetric whereas the Low and High functions are asymmetric in the extremes.

In the first step, given a set of *n *expressed sequence tags (ESTs) or genes belonging to *m *microarrays, the discretization process is based on determining the membership function of each gene to the previously linguistic labels. In this package, two types of membership functions are used (see additional file 1:MembershipFunctions.pdf for more details about the mathematical background). Firstly, a polynomial approximation of a Gaussian membership function which achieve smoothness for the degree of membership of 'normal' expression levels of a gene, and secondly, a polynomial approximation of two sigmoidal membership functions which are able to specify asymmetric membership functions for the 'low' and 'high' expression levels (see Figure 1).

The algorithm defines a threshold value θ, which need to be established in order to discretize the original data in a binary way. For concrete values of threshold θ, specific zones of the gene values domain for which none of the labels will be activated can exist (neighbor region of the intersection of labels Medium and High in Figure 1). This fact must be interpreted as the specific value of the gene is not enough to assign it a significant linguistic label at the significance degree of membership fixed by threshold θ.

On the other hand, one expression level can simultaneously activate two linguistic labels, since at the significance level given by θ, any assignment of the measure to a linguistic label is significant (neighbor region of the intersection of labels Low and Medium in Figure 1).

### Assembling a supervised fuzzy pattern of representative genes

A fuzzy pattern is a higher concept built from a set of FMDs belonging to the same class, and it can be viewed as a prototype of them. The FP corresponding to a given class is constructed by selecting the genes with a label which has a relative frequency of appearance equal to or greater than a predefined ratio π (0 < π ≤ 1). Therefore, the FP captures relevant and common information about the discretized gene expression levels of the FMDs that summarizes.

The predefined ratio π controls the degree of exigency for selecting a gene as a member of the pattern, since the higher the value of π, the fewer the number of genes which make up the FP. The pattern's quality of fuzziness is given by the fact that the labels, which make it up, come from the linguistic labels defined during the transformation into FMD of an initial observation. Moreover, if a specific label of a gene is very common in all the examples belonging to a given class, this feature will be selected to be included in the FP. Therefore, a frequency-based criterion is used for selecting a gene as part of the fuzzy pattern.

### Recognizing valuable genes

The goal of gene selection is to determine a reduced set of genes, which are meaningful given the existing knowledge. Here, the algorithm introduces the notion of discriminant fuzzy pattern with regard to a collection of FPs. A DFP version of a FP only includes those genes that can serve to differentiate it from the rest of the patterns. Therefore, the computed DFP for a specific FP is different depending on what other FPs are compared with it. It's not surprising that the genes used to discern a specific class from others (by mean of its DFP) will be different if the set of rival classes also changes. The pseudo code algorithm used to compute the final DFP containing the selected genes can be consulted in additional file 2:DFPpseudocode.pdf.

## Results and discussion

The package DFP has been designated for performing fuzzy analysis and gene reduction from a set of microarray experiments. DFP, like any R package, is command-line driven. The functions are called by the user, possibly with arguments and options. Any session using DFP in R starts with the command

library (DFP)

which makes the functions of DFP available in the R environment.

A very quick start example could be carried out using the artificial data set rmadataset, included in the package

data(rmadataset)

Once the data is loaded, the whole algorithm can be executed calling its main function discriminantFuzzyPattern(rmadataset) which will work out with the default parameter values, or step by step as in the following example

mfs<-calculateMembershipFunctions

**Figure 2:**
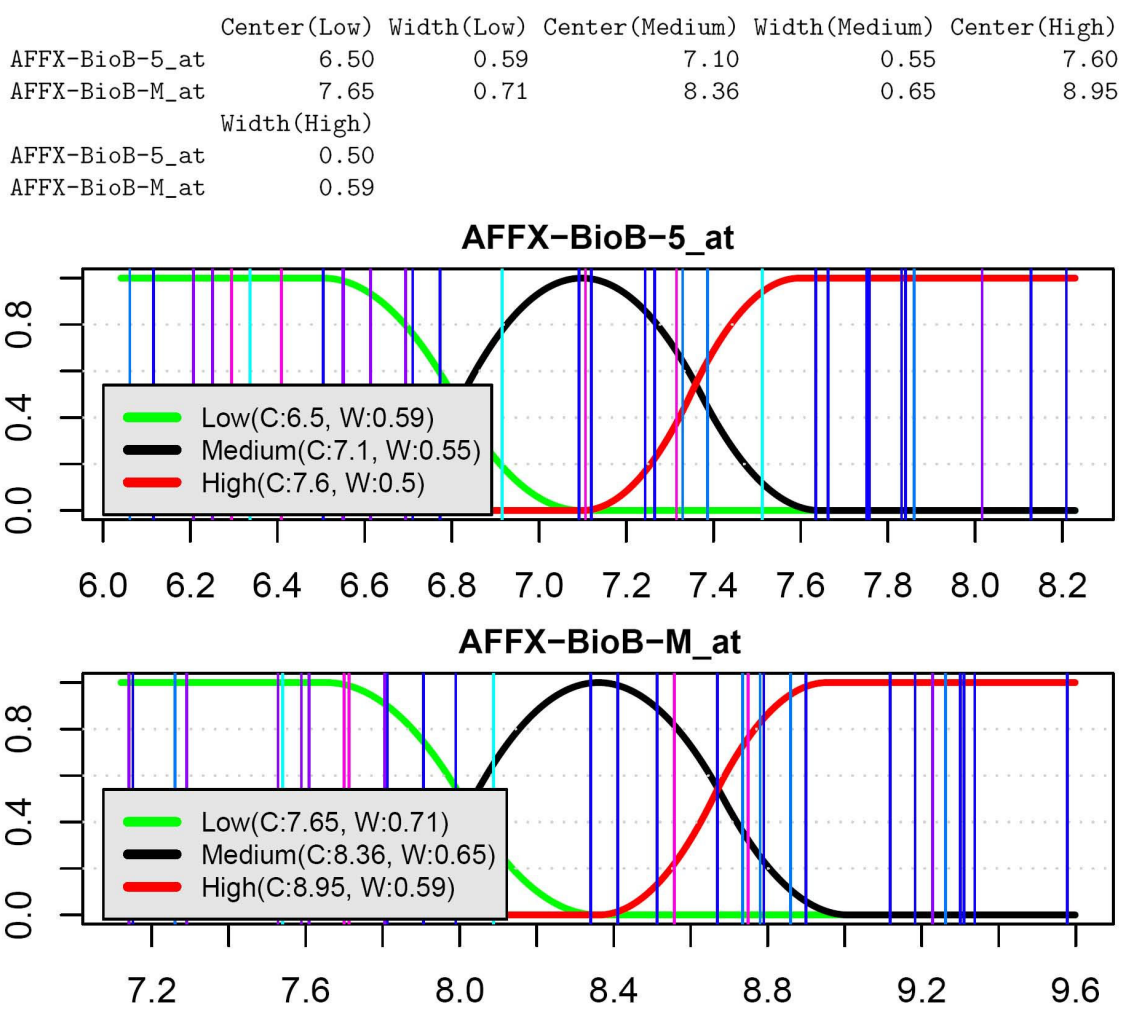
**Membership functions belonging to the first two genes**. Vertical lines show the expression values corresponding to each microarray sample.

+                              (rmadataset, skipFactor = 3)

which calculates the membership functions (Low, Medium, High) for each gene. These functions can be displayed using the following command (see Figure 2)

plotMembershipFunctions

+         (rmadataset, mfs, featureNames(rmadataset [1:2])

DFP can now convert gene expression values (raw data) into linguistic labels. A gene will have an assigned linguistic label if its expression level exceeds the significance degree of membership fixed by threshold zeta (θ). It is done by the command

dvs<-discretizeExpressionValues

+              (rmadataset, mfs, zeta = 0.5, overlapping = 2)

showing part of the results with the following function

showDriscreteValues

+                   (dvs, featureNames(rmadataset) [1:10],

+                               c("healthy", "AML-inv")))

The next step involves the generation of a fuzzy pattern that summarizes the most relevant genes of each category. A gene will belong to a FP if its assigned label is present with a frequency higher than piVal (π). It is done by the command

fps<-calculateFuzzyPatterns

+               (rmadataset, dvs, piVal = 0.9, overlapping)

showing part of the results with the following function

showFuzzyPatterns (fps, "healthy") [21:50]

The last step calculates the discriminant fuzzy pattern by including those genes present in two or more fuzzy patterns with different assigned labels. The following command performs this operation

dfps<-calculateDiscriminantFuzzyPattern (rmadataset, fps)

**Figure 3:**
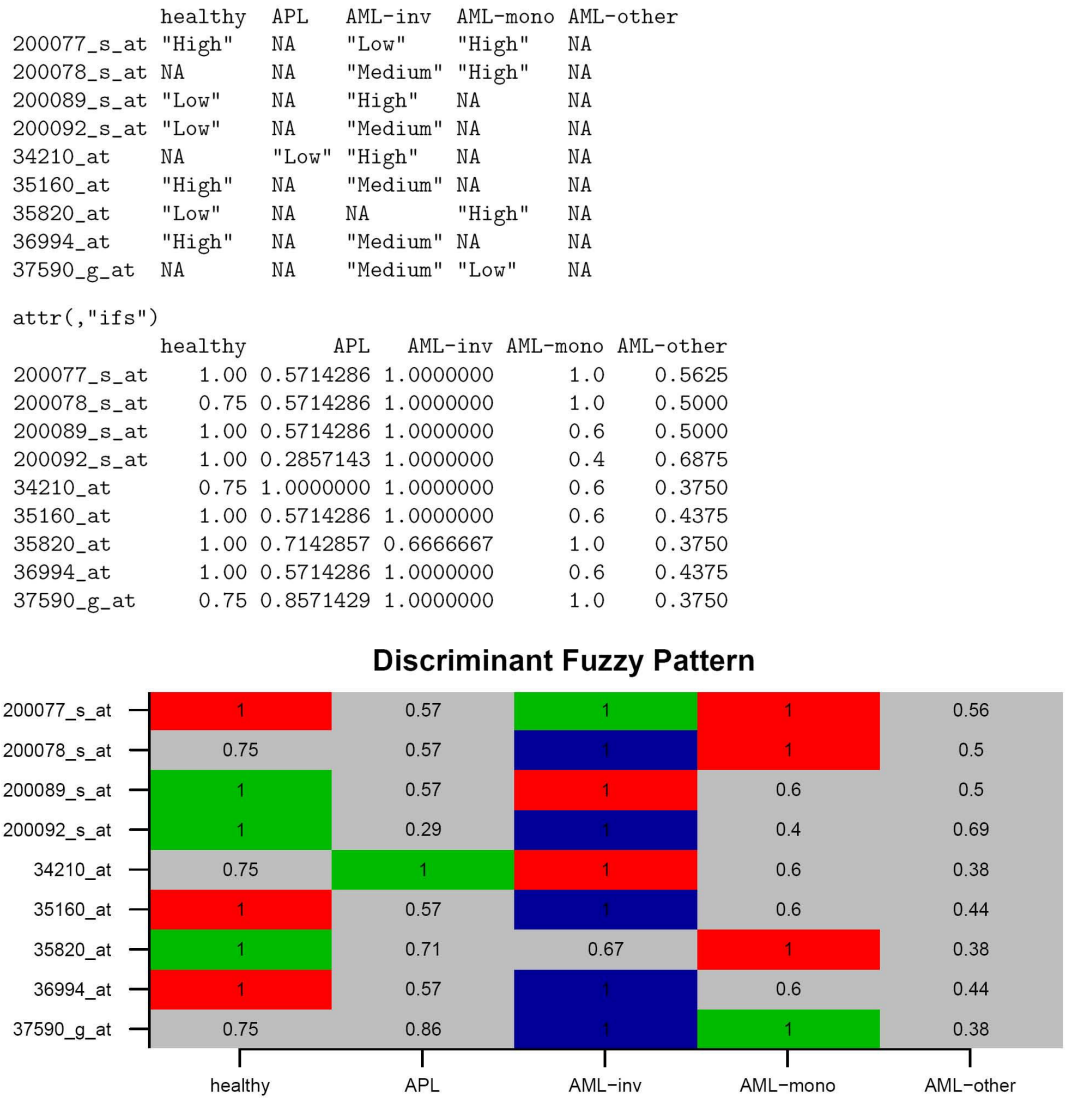
**DFP of selected genes (in rows) with its appearance frequency for each category (in columns)**. In the first table, a NA value is assigned if the frequency of appearance is lower or equal than the piVal parameter, meaning that this gene does not belong to the FP of this category.

The selected genes can now be shown in both text and graphical mode (see Figure 3) using the function

plotDiscriminantFuzzyPattern(dfps, overlapping = 2)

## Conclusion

DFP is a new Bioconductor R package that performs gene selection and data reduction by the application of a supervised fuzzy pattern algorithm. As other Bioconductor/R packages, DFP offers a high level of standardized documentation through its vignette and the function help pages.

The implemented algorithm has also been coded and tested in GENECBR, a multiplatform open source tool for microarray analysis [18]. The results obtained using publicly available data sets validate the effectiveness of the proposed algorithm [19].

## Availability and requirements

**Project name**: DFP

**Project home page**: 

**Operating systems**: Platform independent

**Programming language**: R

**Other requirements**: R, Bioconductor

**License**: GNU GPL

## Authors' contributions

DGP and FFR programmed and tested geneCBR application. RA and FD implemented and tested the code of the DFP package. FFR wrote the paper while DGP, RA and FD provided comments and discussion. All authors read and approved the final manuscript.

## Supplementary Material

Additional file 1 **Definition of Gaussian membership functions implemented in the DFP package.** The membership functions to linguistic labels are defined in a similar way to the form that has been used by Pal and Mitra (2004) [doi:10.1109/TKDE.2003.1262181]. These authors used a polynomial function that approximates a Gaussian membership function, where its centre and amplitude depend on the mean and on the variability of the available data respectively. The original membership functions are considered symmetric, but, in our work we have considered asymmetric functions for the linguistic labels in the extremes (labels Low and High). Click here for file

Additional file 2 **Pseudo code algorithm used to compute the final DFP containing the selected genes.** A DFP version of a FP only includes those genes that can serve to differentiate it from the rest of the fuzzy patterns. Click here for file
