# Supplementary material for: DFP: a Bioconductor package for fuzzy profile identification and gene reduction of microarray data
Source: BMC Bioinformatics. 2009 Jan 29;10:37. doi: 10.1186/1471-2105-10-37 (PMC2637236; doi:10.1186/1471-2105-10-37)
Supplement: Additional file 1 — Definition of Gaussian membership functions implemented in the DFP package. The membership functions to linguistic labels are defined in a similar way to the form that has been used by Pal and Mitra (2004) [doi:10.1109/TKDE.2003.1262181]. These authors used a polynomial function that approximates a Gaussian membership function, where its centre and amplitude depend on the mean and on the variability of the available data respectively. The original membership functions are considered symmetric, but, in our work we have considered asymmetric functions for the linguistic labels in the extremes (labels Low and High). [file 1471-2105-10-37-S1.pdf]

Membership function for the label LOW (asymmetric):

$$\mu_{jL}(x)=\begin{cases}1 & \text{if } x-c_{jL}\le 0\\ 1-2\left(\dfrac{x-c_{jL}}{\lambda_{jL}}\right)^2 & \text{if } 0\le x-c_{jL}\le\dfrac{\lambda_{jL}}{2}\\ 2\left(1-\dfrac{x-c_{jL}}{\lambda_{jL}}\right)^2 & \text{if } \dfrac{\lambda_{jL}}{2}\le x-c_{jL}\le\lambda_{jL}\\ 0 & \text{otherwise}\end{cases} \quad (1)$$

where c_{jL} is the mean of the values of feature F_j below the mean of all values of the feature F_j, (namely, given $c_{jM} = \mathrm{E}[F_j]$, the centre c_{jL} is the mean of the values of feature F_j that are comprised between $\mathtt{min}(F_j)$ and c_{jM}) and the λ_{jL} parameter is the distance between c_{jM} and c_{jL}, $\lambda_{jL} = c_{jM} - c_{jL}$.

Membership function for the label HIGH (asymmetric):

$$\mu_{jH}(x)=\begin{cases}1 & \text{if } x-c_{jH}\ge 0\\ 1-2\left(\dfrac{x-c_{jH}}{\lambda_{jH}}\right)^2 & \text{if } -\dfrac{\lambda_{jH}}{2}\le x-c_{jH}\le 0\\ 2\left(1+\dfrac{x-c_{jH}}{\lambda_{jH}}\right)^2 & \text{if } -\lambda_{jH}\le x-c_{jH}\le-\dfrac{\lambda_{jH}}{2}\\ 0 & \text{otherwise}\end{cases} \quad (2)$$

where the centre c_{jH} is the mean of the values of F_j that are comprised between the mean value of all values, c_{jM}, and the maximum value, $\mathtt{max}\{F_j\}$, whereas the amplitude parameter, λ_{jH}, is given by the difference $c_{jH} - c_{jM}$. This function extends the right side of the domain of Feature F_j.

Membership function for the label MEDIUM (symmetric):

$$\mu_{jM}(x)=\begin{cases}1-2\left(\dfrac{\|x-c_{jM}\|}{\lambda_{M}}\right)^2 & \text{if } 0\le\|x-c_{jM}\|\le\dfrac{\lambda_{jM}}{2}\\ 2\left(1-\dfrac{\|x-c_{jM}\|}{\lambda_{jM}}\right)^2 & \text{if } \dfrac{\lambda_{M}}{2}\le\|x-c_{jM}\|\le\lambda_{jM}\\ 0 & \text{otherwise}\end{cases} \quad (3)$$

where the centre parameter, c_{jM}, is the mean of all values of feature F_j, $c_{jM} = \mathrm{E}[F_j]$, and the amplitude parameter λ_{jM} is given by the half of the distance between the centres of the extreme functions, namely, $\lambda_{jM} = ½ (c_{jH} - c_{jL})$.
