# Supplementary material for: DFP: a Bioconductor package for fuzzy profile identification and gene reduction of microarray data
Source: BMC Bioinformatics. 2009 Jan 29;10:37. doi: 10.1186/1471-2105-10-37 (PMC2637236; doi:10.1186/1471-2105-10-37)
Supplement: Additional file 2 — Pseudo code algorithm used to compute the final DFP containing the selected genes. A DFP version of a FP only includes those genes that can serve to differentiate it from the rest of the fuzzy patterns. [file 1471-2105-10-37-S2.pdf]

Pseudo code algorithm used to compute the final DFP:

```
procedure DiscriminantFuzzyPatterns (input: ListFP; output: ListDFP)
{
begin
  initialize_DFP: FP ← ∅
  for each fuzzy pattern FPi ∈ ListFP do
    Initialize_DFP: DFPi ← ∅
    for each fuzzy pattern FPj ∈ ListFP and FPi <>FPj do
      for each gen g ∈ GetGenes(FPi) do
        if   (g ∈ GetGenes(FPj)) AND
             (GetLabel(FPi, g) <> GetLabel(FPj, g)) then
               AddMember(DFPi, Member(FPi, g))
    Add_to_List_of_DFP: Add(ListDFP, DFPi)
end.
}
```
